# Returns After Discharge From the Emergency Department Observation Unit: Who, What, When, and Why?

**DOI:** 10.5811/westjem.59023

**Published:** 2023-05-03

**Authors:** Daniel Berger, Steven King, Catherine Caldwell, Erik F. Soto, Andrew Chambers, Susan Boehmer, Ravindra Gopaul

**Affiliations:** * Pennsylvania State University College of Medicine, Hershey, Pennsylvania; † Penn State Health Milton S. Hershey Medical Center, Department of Emergency Medicine, Hershey, Pennsylvania

## Abstract

**Introduction:**

The number of emergency department observation units (EDOU) and observation stays has continued to increase. Despite this, there is limited data on the characteristics of patients who return unexpectedly to the ED after EDOU discharge.

**Methods:**

We identified the charts of all patients who were admitted to the EDOU of an academic medical center between January 2018–June 2020 and had a return to the ED within 14 days of discharge from the EDOU. Patients were excluded if they were admitted to the hospital from the EDOU, left against medical advice, or died in the EDOU. We manually extracted selected demographic factors, comorbidities, and healthcare utilization data from the charts. Physician reviewers identified return visits thought to be related to the index visit or potentially avoidable.

**Results:**

During the study period, there were 176,471 ED visits, 4,179 admissions to the EDOU, and 333 return visits to the ED within 14 days from discharge from the EDOU, representing 9.4% of all patients discharged from the EDOU. We identified a higher rate of return for patients treated for asthma and lower rates of return for patients treated for chest pain or syncope than the overall return rate. Physician reviewers determined that 64.6% of unplanned returns were related to the index visit, and 4.5% were potentially avoidable. Of potentially avoidable visits, 53.3% occurred within 48 hours of discharge, supporting the use of this period as a potential quality metric. While there was no significant difference in the percentage of related return visits between males and females, there was a higher rate of potentially avoidable visits for male patients.

**Conclusion:**

This study adds to the limited body of literature on EDOU returns, finding an overall return rate of under 10%, with about two-thirds of returns determined to be related to the index visit and <5% considered to be potentially avoidable.

## INTRODUCTION

Emergency department observation units (EDOU) provide outpatient observation services for patients who do not meet inpatient criteria but still require additional care before they can be safely discharged from the ED. These units have an average length of stay (LOS) of 10 hours per patient and are capable of caring for 5–10% of ED volume.^1^ On average, 80% of EDOU patients can be safely discharged, while the remaining 20% will be upgraded to inpatient status.^1^

**Figure 1:**
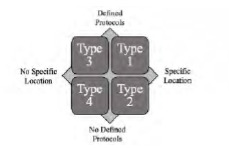
Types of emergency department observation units.

There are four types of observation units (Figure 1). Type 1 units are the most structured, with care governed by specific protocols and provided within a designated area. Type 2 units use a designated area but do not have specific protocols. Type 3 units use specific protocols but lack a designated observation area. Type 4 units lack both protocols and a designated area.^2^ Type 1 units have been shown to perform best, resulting in shorter LOS, lower rates of admission, and better clinical outcomes.^2^ EDOUs have also been reported to improve patient satisfaction.^3^

While EDOUs have existed since the 1960s, the number of observation stays resulting from ED visits has significantly grown.^2^ From 2001 to 2008, observation stays increased over 360%, from 0.6% of ED visits in 2001 to 1.9% in 2008.^4^ By 2008, over 34% of EDs had an EDOU, 56% of which were under ED administrative control.^4^ Observation units are associated with reduced cost, with a 27–42% lower cost in a Type 1 unit compared to a similar inpatient stay.^2^,^5^ A 2012 study estimated that if all hospitals had an EDOU, over 2.4 million inpatient visits could be avoided, saving 3.1 billion dollars annually.^5^ If these units were all Type 1 units, potential savings could be up to 8.5 billion dollars annually.^2^

However, there is limited research about the rate of unplanned returns to the ED (colloquially known as “bouncebacks”) of patients discharged from observation units. Although some studies have analyzed return visits for specific conditions, few have examined overall rates of return or compared the return rates for different complaints. The primary outcome of this paper was to describe demographic characteristics and complaints associated with higher rates of return compared to EDOU rates at large. Secondary outcomes included approximate time-to-return for return visits that were related to the initial EDOU stay or considered potentially avoidable.

## METHODS

The charts of all patients admitted to the EDOU of an academic medical center between January 2018 –June 2020 were exported from the electronic health record (Cerner Corporation, Kansas City, MO) into Microsoft Excel (Microsoft Corporation, Redmond, WA). Our Type 1 EDOU is staffed with advanced practice providers (APP) and supervised by an attending physician. A list of EDOU protocols is available in Appendix 1. All patients who returned to the ED within 14 days of discharge from the EDOU were identified. We excluded patients if they were admitted to the hospital from the EDOU, left against medical advice, or died in the EDOU.

**Population Health Research Capsule**

What do we already know about this issue?

*Use of emergency department observation units (EDOU) is increasing. However, research on unplanned returns to the ED after discharge is limited*.

What was the research question?

*We aimed to identify patient demographics or diagnoses associated with higher rates of return after discharge*.

What was the major finding of the study?

*Our return rate was 9.42% (CI 8.45–10.38%), with 64.6% of returns related to the index visit and 4.5% potentially avoidable*.

How does this improve population health?

*Greater understanding of rates and reasons for return visits can inform how to reduce unplanned returns after discharge from the EDOU*.

Selected demographic factors, comorbidities, and healthcare utilization data were manually extracted from the charts. The EDOU medical director then sorted the data into categories by treatment protocol. When patients had multiple complaints, they were categorized under the primary complaint protocol. Once charts were sorted, two blinded emergency physicians reviewed the patients’ charts. Using their clinical judgment, they determined whether the return ED visit was related to the original EDOU visit (i.e., the same complaint) and whether it could have potentially been avoided by actions taken during the EDOU admission. A third physician reviewed and adjudicated any disagreements between the other reviewers.

A report was generated for all EDOU patients containing each visit’s diagnosis and treatment plan. We manually coded each unique pairing into the appropriate treatment protocol category, with codes then applied in bulk to the duplicate pairings. We used visits grouped by EDOU protocol when calculating the rate of related and potentially avoidable visits, whereas visits sorted by diagnosis were used to calculate return rates by complaint. Adult and pediatric patients were split into subpopulations, as different protocols were used for patients <18 years. Additionally, we compiled a report of the age and gender of all patients treated in the ED during the same period. The remaining ED and EDOU records for patients who did not return during the study period served as a comparison population.

We performed statistical analysis using SAS version 9.4 (SAS Institute Inc., Cary, NC). We generated descriptive statistics and used chi-square and Fisher exact tests to identify statistically significant differences within the return population. For each complaint, we calculated the rate of return with 95% confidence interval (CI).

## RESULTS

### ED and EDOU Visits

**Figure 2:**
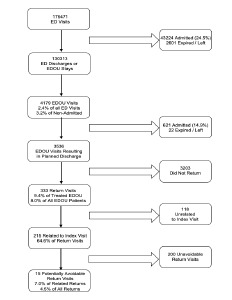
Workflow to determine emergency department (ED), emergency department observation unit (EDOU), and return visits for inclusion.

Between the opening of the EDOU in January 2018 and the time of data collection in June 2020, there were 176,471 ED visits, of which 43,224 (24.5%) resulted in hospital admissions. A total of 2,289 (1.3%) patients left against medical advice or without being seen; 312 (0.2%) patients died; 126,134 (71.5%) were discharged; and 4,179 (2.4%) were admitted to the EDOU. Of the 4,179 EDOU visits, 621 (14.9%) patients were admitted to the hospital, 21 (0.5%) left during treatment or against medical advice, one died, and 3,536 (84.6%) were successfully treated and discharged. Of those 3,536 patients, 333 had a return visit to the ED within 14 days of discharge from the EDOU, representing 9.4% of all patients discharged from the EDOU and 8.0% of all patients ever admitted to the EDOU. Of these 333 return visits, 215 (64.6%) were determined by two physician-reviewers to be related to the index visit and 15 (7.0% of related returns, 4.5% of all returns) were determined to have been potentially avoidable. A flowchart outlining this process is shown in Figure 2.

### Rate of Returns

The overall rate of returns was 9.42% (CI 8.45–10.38%). The return rate among adult patients was 9.74% (CI 8.72–10.76%), compared with 5.67% (CI 3.28–8.37%) among pediatric patients. Table 1 shows the most common adult complaints for EDOU admission and the return rate for each complaint. The most common reasons for adult EDOU admissions were for chest pain (18.9%), cellulitis (11.2%), dehydration (9.3%), and abdominal pain (7.4%). The rate of return for patients treated for asthma (17.5%; CI 10.7–24.3%) was higher than the overall return rate. The rate of return for patients treated for chest pain (6.5%; CI 4.6–8.3%) or syncope (5.1%; CI 2.2–8.2%) was lower than the overall return rate. A complete list of adult return rates is available in Appendix 2.

The most common complaints treated in the EDOU for pediatric patients were bronchiolitis (19.4% of pediatric EDOU patients; 1.5% of all EDOU patients), dehydration (17.8% of pediatric EDOU patients; 1.4% of all EDOU patients), and asthma (17.2% of pediatric EDOU patients; 1.3% of all EDOU patients) (Table 2). There were no pediatric return rates for any specific complaint greater than the overall pediatric return rate. No pediatric patients who were treated for abdominal pain (10) or pyelonephritis (6) returned during the study period. A complete list of pediatric return rates is available in Appendix 2.

### Characteristics of the Return Population

The study population was overwhelmingly White (86.2%) and English-speaking (97%). Compared with males, females were less likely to be married (36.8% vs 53.1%; *P=*.003) and more likely to be separated or divorced (24.5% vs 16.4%; *P*=0.01) or widowed (12.3% vs 3.1%, *P*<.001). Females were also more likely to arrive at the ED by ambulance (*P*=.01). Males were more likely to use tobacco (25.0% vs 12.7%; *P*<.001) and alcohol (23.8% vs 12.1%; *P*=.007) and have aspirin in their medication list (31.7% vs 21.6%, *P*=.047). There were no differences in gender by insurance type (*P*=0.22), hospitalizations (*P*=0.23), additional ED visits (beyond index visit and return visit; *P*=.10), or primary care physician visits (*P*=0.96) during the time between the index EDOU stay and their return visit. Demographic information is shown in Appendix 3.

### Timing of Returns

Overall, 14.4% of returns occurred within 24 hours, 27.3% within 48 hours, and 65.76% within 72 hours, with similar timing of returns for males and females. Although only 30.2% of related visits occurred within 48 hours of discharge, 53.3% of potentially avoidable visits occurred during this period.

### Related and Potentially Avoidable Visits

Physician reviewers agreed that 215 of the 333 return visits (64.6%) were related to the initial visit and 15 of 332 (4.5%) return visits were potentially avoidable. While there was no significant difference between male and female patients in the percentage of return visits that were related to the original visit (69.5% vs 61.5%; *P*=0.13), there was a significantly higher rate of potentially avoidable visits among males (8.7% vs 2.0%, *P*=.004). Reasons for potentially avoidable return visits included medications issues (errors in prescription or patient was unable to obtain), incomplete workup, lack of specialist consultation, or reviewers believed that the patient should have been admitted to the hospital during the initial ED visit.

### Visits by Gender

When comparing the percentage of female patients in the ED population with that of the EDOU, there was no significant difference (56.3% vs. 54.9%; *P*=0.12). Although there was a higher percentage of females in the return visit population than in the ED populations (61.6% vs 54.9%; *P*=.02), there was no significant difference between the percentage of females in the EDOU and return visit populations (56.3% vs 61.6%; *P*=.07).

### Length of Stay

The overall population had a mean LOS of 26.89±11.52 hours in the ED and a mean LOS of 20.55±11.49 in the EDOU. There was no statistically significant difference t(332)=0.66, *P*=0.5 between the LOS in the ED for male (mean [M] 26.36, SD 13.45) and female (M 27.21, SD 10.16) patients, nor the EDOU LOS t(332)=0.44, *P*=0.6 for male (M 20.20, SD 13.87) and female (M 20.77, SD 9.75) patients. Patients who did not have a return visit had a mean EDOU LOS (M 13.00, SD 6.27).

### Visits by Age

The mean age of patients who returned was 56.21 years (CI 53.77–58.65), not statistically different from the mean age of 54.32 years (CI 53.47–55.18) of patients who did not return. There was also no difference between the ages of males and females in the return population, nor between each respective gender when compared to the population that did not return. A complete list of mean ages for the subgroups of the populations with and without return visits are available in Appendix 4.

## DISCUSSION

Our results are similar to those reported in two previous studies of academic EDOUs conducted by Ross et al. and Southerland et al.^6^,^7^ In addition to having a similar average age and percentage female, we found no statistically significant difference between the return rates of males and females and the makeup of the EDOU at large.^6^ While our EDOU’s 14.9% hospital admission rate was somewhat lower than the 19% and 23.5% reported by Ross and Southerland, respectively, we found percentages of EDOU patients who returned similar to those reported by Ross (9.4% vs 10.7%).^6^,^7^ Our rate of returns related to the initial visit was also similar to that found in the Ross study (65% vs 74%).^6^ We were unable to locate any previous studies that attempted to determine whether the EDOU return visits were potentially avoidable.

When comparing between males and females, there was no significant difference in LOS. This is in line with prior research that examined the LOS in observations units.^8^–^9^ Previous studies have demonstrated that LOS is usually associated with factors beyond the ED’s control, including organizational factors.^10^ Other studies have suggested that triage level, consultations, and investigative testing are causes for prolonged LOS.^11^

While the majority of complaints had a return rate that was not significantly different from our overall return rate, our return rate for adults treated for asthma was 17.5%. This was not only higher than our overall return rate, but higher than the 12.1% of asthma patients who returned to the ED within one year of an ED visit and the 30-day readmission rate for hospitalized asthma patients of 11.9% reported in prior literature.^12^,^13^ However, a previous study of EDOU asthma returns found a rate of 9%, suggesting that our EDOU may accept a higher acuity of asthma patients or indicate the need to refine our treatment protocol.^6^ We also found lower rates of return for patients treated for chest pain and syncope, indicating these patients are well suited for EDOU care. Previous research also supports findings of lower rates of chest pain returns from the EDOU.^6^

Although less than one-third of related visits occurred within 48 hours of discharge, more than half of potentially avoidable visits occurred during this period. This suggests that using a 48-hour window for quality review might catch a majority of potentially avoidable visits, allowing for development of protocol improvements that could reduce return visits. It is important to note that for visits determined to be potentially avoidable, it does not necessarily mean there was a medical error. Our physician review team conducted a thorough review of the patient records, something that may not have been possible or indicated at the original ED visit. In some situations, consults or further workup may have been deferred because of the clinical status of the patient or patient preference, items that may not have been documented in the chart.

Our EDOU study population was very large and comprised of data collected over a 30-month period, enabling generation of an overall return rate with a relatively narrow CI, as well as generation of individual return rates for primary complaints. Our overall return rate and return rate of related visits were similar to those of a previous study, supporting the validity of our results. However, in our study, we went further by attempting to quantify the rate of potentially related return visits.

## LIMITATIONS

We acknowledge that this study has several limitations. First, it took place at a single academic medical center whose patient population was overwhelmingly White, English-speaking, and insured, potentially limiting generalizability to differing populations. Although Type 1 EDOU units are well defined, differences in staffing, primary caregiver (physician vs APP), capabilities of the unit, types of protocols, and overall efficacy of institutional treatment protocols could pose further barriers to generalizability to other institutions. Second, patients who had a return visit to hospitals outside the health system would not have been captured in the study, potentially yielding a lower return rate than the true rate. Patients who returned after day 14 from EDOU discharge were not included in the study, also potentially affecting the true return rate.

The study period also included the first three months of the COVID-19 pandemic in the US. During this period, many hospitals saw a reduction in patients, potentially affecting our return rate.^14^ Lastly, although multiple physician-reviewers were used to categorize visits as related or avoidable, what is considered avoidable is highly subjective and varied between reviewers. Additionally, the reviewers knew that the charts they were reviewing were from patients who had a return visit; so their attributions may have been affected by hindsight bias. For example, after a records review, one reviewer noted that although a patient’s vital signs were within normal range, they were abnormal for the patient in question. While this was factually correct, it is unlikely that the treating physician would have reached this conclusion while caring for the patient without an indication to conduct an extensive chart review. Future studies should establish criteria for what visits could “reasonably” be avoided.

## CONCLUSION

This study adds to the limited body of literature on returns to ED observation units, finding an overall return rate of under 10%, with about two-thirds of returns determined to be related to the index visit and <5% considered to be potentially avoidable. Our study demonstrates findings consistent with previous single-center studies, including return rates. In addition, this study demonstrates that potentially avoidable revisits were likely to occur within the first 48 hours of discharge. Additional studies should include data from multiple institutions and further explore returns related to potentially avoidable revisits.

## Supplementary Information



## Figures and Tables

**Table 1:** Most commonly used adult protocols in the emergency department observation unit.

Complaint	N (%) of EDOU admissions	Return visits N (%; 95% CI)
Chest pain	667 (18.86)	43 (6.45%; 4.58–8.31)
Cellulitis	395 (11.17)	35 (8.86%; 6.06–11.66)
Dehydration	330 (9.33)	33 (10.00%; 6.76–13.24)
Abdominal pain	262 (7.41)	33 (12.60%; 8.58–16.61)
Ambulatory dysfunction	234 (6.62)	27 (11.54%; 7.44–15.63)
Syncope	196 (5.54)	10 (5.10%; 2.20–8.18)
UTI	139 (3.93)	13 (9.35%; 4.51–14.19)
Asthma	120 (3.39)	21 (17.5%; 10.7–24.3)
Anemia	104 (2.94)	7 (6.73%; 1.92–11.55)
Dizziness	97 (2.74)	5 (5.15%; 0.75–9.55)
GI bleed	96 (2.71)	10 (10.42%; 4.31–16.53)

*CI*, confidence interval; *EDOU*, emergency department observation unit; *GI*, gastrointestinal; *UTI*, urinary tract infection.

**Table 2:** Most commonly used pediatric protocols in the emergency department observation unit.

Complaint	N (%) of EDOU admissions	Return visits N (%; 95% CI)
Bronchiolitis	52 (1.47)	2 (3.85%; 0.00–9.07)
Dehydration	48 (1.36)	1 (2.08%; 0.00–6.12)
Asthma	46 (1.30)	1 (2.17%; 0.00–6.39)
Soft tissue infection	40 (1.13)	4 (10.00%; 0.70–19.30)
Croup	31 (0.88)	5 (16.13%; 3.18–29.08)

*CI*, confidence interval; *EDOU*, emergency department observation unit; *CI*, confidence interval.
