# Supplementary material for: Returns After Discharge From the Emergency Department Observation Unit: Who, What, When, and Why?
Source: West J Emerg Med. 2023 May 3;24(3):390–5. doi: 10.5811/westjem.59023 (PMC10284506; doi:10.5811/westjem.59023)
Supplement: Supplementary file 1 [file wjem-24-390-s001.docx]

**Appendix 1: List of Emergency Department Observation Unit Protocols**

**Adult EDOU Protocols**

Abdominal Pain

Accidental Overdose/ETOH Intoxication

Acute Back Pain

Atrial Fibrillation

Allergic Reaction/Angioedema

Ambulatory Dysfunction

Anemia

Asthma/COPD

Cellulitis

Chest Pain

Clostridium Difficile

Congestive Heart Failure

Dehydration

Dizziness/Vertigo

DVT (Upper and Lower extremity)

Electrolyte Abnormalities (K, Ca, Na, MG)

Gastrointestinal Bleed

Headache/Migraine

Hyperemesis (early pregnancy)

Hyperglycemia

Influenza

Joint Pain/Septic Joint/Osteomyelitis

Opioids

Pharyngitis

Pneumonia

Procedures

Pulmonary Embolism

Renal Colic

Seizure

Syncope

Transient Neuro Deficits/Stroke-like Symptoms

Urinary Tract Infection/Pyelonephritis

**Pediatric EDOU Protocols**

Abdominal Pain

Accidental Ingestion

Anaphylaxis

Asthma

Bronchiolitis

Croup

Dehydration

Drowning

Influenza

Pharyngitis

Pneumonia

Pyelonephritis

Soft Tissue Infection

**Appendix 2: Return Rates by Complaint**

**Adult Return Rates by Complaint**

|  | # EDOU (% of EDOU) | # Returns (% Rate of Return) | % Return Rate CI |
| --- | --- | --- | --- |
| Abdominal Pain | 262 (7.41) | 33 (12.60) | (8.58 - 16.61) |
| Accidental Overdose | 17 (0.48) | 1 (5.88) | (0 - 17.07) |
| Acute Back Pain | 80 (2.26) | 9 (11.25) | (4.33 - 18.17) |
| Allergic Reaction | 39 (1.10) | 6 (15.38) | (4.06 - 26.71) |
| Ambulatory Dysfunction | 234 (6.62) | 27 (11.54) | (7.44 - 15.63) |
| Anemia | 104 (2.94) | 7 (6.73) | (1.92 - 11.55) |
| Asthma* | 120 (3.39) | 21 (17.50) | (10.70 - 24.30) |
| Atrial Fibrillation | 15 (0.42) | 2 (13.33) | (0.00 - 30.54) |
| Cellulitis | 395 (11.17) | 35 (8.86) | (6.06 - 11.66) |
| Chest Pain* | 667 (18.86) | 43 (6.45) | (4.58 - 8.31) |
| Clostridium Difficile | 4 (0.11) | 2 (50.00) | (1.00 - 99.00) |
| Congestive Heart Failure | 41 (1.16) | 7 (17.07) | (5.56 - 28.59) |
| Dehydration | 330 (9.33) | 33 (10.00) | (6.76 - 13.24) |
| Dizziness | 97 (2.74) | 5 (5.15) | (0.75 - 9.55) |
| Deep Vein Thrombosis | 26 (0.74) | 5 (19.23) | (4.08 - 34.38) |
| Electrolyte Abnormalities | 57 (1.61) | 9 (15.79) | (6.32 - 25.26) |
| Gastrointestinal Bleed | 96 (2.71) | 10 (10.42) | (4.31 - 16.53) |
| Headache/Migraine | 24 (0.68) | 4 (16.67) | (1.76 - 31.58) |
| Hyperemesis Gravidum | 15 (0.42) | 1 (6.67) | (0.00 - 19.29) |
| Hyperglycemia | 35 (0.99) | 3 (8.57) | (0.00 - 17.85) |
| Hypertension | 1 (0.03) | 0 (0.00) |  |
| Influenza | 20 (0.57) | 1 (5.00) | (0.00 - 14.55) |
| Joint Pain | 20 (0.57) | 2 (10.00) | (0.00 - 23.15) |
| Opioids | 6 (0.17) | 0 (0.00) |  |
| Pharyngitis | 18 (0.51) | 3 (16.67) | (0.00 - 33.88) |
| Pneumonia | 54 (1.53) | 6 (11.11) | (2.73 - 19.49) |
| Procedures | 21 (0.59) | 6 (28.57) | (9.25 - 47.89) |
| Pulmonary Embolism | 10 (0.28) | 2 (20.00) | (0.00 - 44.79) |
| Renal Colic | 44 (1.24) | 7 (15.91) | (5.10 - 26.72) |
| Seizure | 12 (0.34) | 1 (8.33) | (0.00 - 23.97) |
| Syncope* | 196 (5.54) | 10 (5.10) | (2.02 - 8.18) |
| Transient Neurolo Deficits | 53 (1.50) | 3 (5.66) | (0.00 - 11.88) |
| Urinary Tract Infection | 139 (3.93) | 13 (9.35) | (4.51 - 14.19) |

**Pediatric Return Rates by Complaint**

| Complaint | # EDOU (% of EDOU) | # Returns (% Rate of Return) | % Return Rate CI |
| --- | --- | --- | --- |
| Abdominal Pain | 10 (0.28) | 0.00 (0.00) |  |
| Accidental Ingestion | 5 (0.14) | 1 (20.00) | (0.00 - 55.06) |
| Anaphylaxis | 4 (0.11) | 1 (25.00) | (0.00 - 67.43) |
| Asthma | 46 (1.30) | 1 (2.17) | (0.00 - 6.39) |
| Bronchiolitis | 52 (1.47) | 2 (3.85) | (0.00 - 9.07) |
| Croup | 31 (0.88) | 5 (16.13) | (3.18 - 29.08) |
| Dehydration | 48 (1.36) | 1 (2.08) | (0.00 - 6.12) |
| Influenza | 15 (0.42) | 0.00 (0.00) |  |
| Pneumonia | 18 (0.51) | 1 (5.56) | (0.00 - 16.14) |
| Pyelonephritis | 6 (0.17) | 0.00 (0.00) |  |
| Soft Tissue Infection | 40 (1.13) | 4 (10.00) | (0.70 - 19.30) |
| Other | 7 (0.20) | 0.00 (0.00) |  |

**Appendix 3: Characteristics of the Population With Return Visits**

| Characteristic | Overall  N (%) | Females  N (%) | Males  N (%) | p-value |
| --- | --- | --- | --- | --- |
| White | 287 (86.2) | 177 (86.3) | 110 (85.9) | 0.917 |
| English Speaking | 323 (97.0) | 197 (96.1) | 126 (98.4) | 0.224 |
| Married | 143 (42.9) | 75 (36.6) | 68 (53.1) | 0.003 |
| Tobacco Use | 58 (17.5) | 26 (12.7) | 32 (25.0) | 0.004 |
| Alcohol Use | 53 (16.6) | 24 (12.1) | 29 (23.8) | 0.007 |
| Drug Use | 27 (8.2) | 5 (2.6) | 4 (3.4) | 0.672 |
| Aspirin Use | 79 (25.5) | 41 (21.6) | 38 (31.7) | 0.047 |
| ≥1 hospitalization within 14 days of EDOU discharge | 67 (20.1) | 37 (18.0) | 30 (23.4) | 0.233 |
| ≥ 1 additional ED visit within 14 days of EDOU discharge | 44 (13.2) | 27 (13.2) | 17 (13.3) | 0.977 |
| ≥ 1 primary care visit within 14 days of EDOU discharge | 42 (12.6) | 26 (12.7) | 16 (12.5) | 0.961 |
| Arrived in an ambulance | 74 (25.8) | 56 (30.8) | 18 (17.1) | 0.011 |
| Type of insurance |  |  |  |  |
| Private | 150 (45.0) | 96 (46.8) | 54 (42.2) | 0.217 |
| Medicare/ MedicareAdvantage | 149 (44.7) | 94 (45.6) | 55 (43.0) |  |
| Medicaid | 4 (1.2) | 3 (1.5) | 1 (0.8) |  |
| Other | 30 (9.0) | 12 (5.9) | 18 (14.1) |  |

**Appendix 4: Mean Age of EDOU and Patients With Return Visits**

|  | No Return Visit | | Return Visit | |
| --- | --- | --- | --- | --- |
|  | Mean Age | CI | Mean Age | CI |
| Overall | 54.32 | 53.47 - 55.18 | 56.21 | 53.77 - 58.65 |
| Female | 55.73 | 54.62 - 56.85 | 56.54 | 53.22 - 59.85 |
| Male | 52.5 | 51.19 - 53.80 | 55.68 | 52.15 - 59.21 |
| Adult | 58.99 | 58.27 - 59.71 | 58.73 | 56.51 - 60.95 |
| Pediatric | 5.04 | 4.45 - 5.65 | 6.25 | 2.66 - 9.84 |
